# In Favor of Controlling Proven, but Not Probable, Causes of Cancer

**DOI:** 10.1289/ehp.1104201

**Published:** 2011-11-01

**Authors:** Thomas C. Erren, J. Valérie Groß, Peter Morfeld

**Affiliations:** Institute and Policlinic for Occupational Medicine, Environmental Medicine and Prevention Research, University Hospital of Cologne, University of Cologne, Cologne, Germany, E-mail: tim.erren@uni-koeln.de; Institute for Occupational Epidemiology and Risk Assessment, Evonik Industries AG, Essen, Germany

We wish to compliment and complement the editorial by Landrigan et al. (2011) who *inter alia* synthesized the “Asturias Declaration” during the “International Conference on Environmental and Occupational Determinants of Cancer: Interventions for Primary Prevention” [World Health Organization (WHO) 2011]. Although the authors list recommendations that are certainly commendable, we strongly disagree with the inclusion of “probable” in the suggestion that “the WHO should develop a global framework for control of environmental and occupational carcinogens that concentrates on the exposures identified by IARC [International Agency for Research on Cancer] as proven or probable causes of human cancer.”

Indeed, we would strongly suggest the need to focus on the causes of human cancer that have been identified by IARC as proven, but not on “probable” causes [59 agents have been classified by IARC as group 2A, i.e., probably carcinogenic to humans (IARC 2011)] to then direct premature prevention efforts on the latter. Soberingly, IARC’s diligent evaluation process of what can and what cannot cause cancer in humans would be blurred when equating group 1 (proven carcinogen) classifications with group 2A classifications, as recommended in the Asturias Declaration. A group 2A classification is not necessarily part of a one-way street to a group 1 verdict.

To provide a recent, empirical example, shift-work that involves circadian disruption was classified as a probable human carcinogen (Straif et al. 2007). Importantly, though, as long as causality is not established, we should clearly be deterred from activities that are not driven by data. Moreover, means for primary prevention are elusive (Erren et al. 2009): Shift-work is unavoidable in our 24/7 societies, and it is impossible with today’s state of knowledge to identify workers who are robust to shift-work conditions and to dissuade others who may be susceptible to the effects of circadian disruption or chronodisruption (Erren et al. 2008; Erren and Reiter 2008). An IARC classification of “probable” human carcinogen, which implies uncertainty and the possibility that future research may exonerate the “culprit in question,” is certainly not an appropriate yardstick to guide valuable and limited resources. Instead, we should invest in controlling established carcinogens such as asbestos and smoking.

Overall, when Richard Nixon declared the war on cancer on 23 December 1971, he remarked, “I hope in the years ahead that we may look back on this day and this action as being the most significant action taken during this administration” (Nixon 1971b). That initiative certainly is not—not only because of the Watergate scandal but, importantly, because of the highly ambitious goal “to find a cure for cancer” (Nixon 1971a). Lacking insights into how to cure cancer in the majority of cases, our objective for now—and presumably for many years to come—should be improved primary prevention of environmentally and occupationally caused cancers. Clearly, although progress in prevention is necessary and feasible, it is imperative to identify realistic and defensible goals and strategies. To this end, a sensible recommendation for strategy would be that “a new global policy framework for environmental cancer” (Landrigan et al. 2011) should focus on established carcinogens such as asbestos, “smoking, overweight, and inactivity” (Willett et al. 2011)—but not on probable culprits.
